# Early Life Trauma and Immune Aging: Evidence of a Biological Threshold in Older Women

**DOI:** 10.1093/geroni/igaf122.2016

**Published:** 2025-12-31

**Authors:** Grace Noppert, Philippa Clarke, Kate Duchowny

**Affiliations:** University of Michigan, Ann Arbor, Michigan, United States; University of Michigan, Ann Arbor, Michigan, United States; University of Michigan, Ann Arbor, Michigan, United States

## Abstract

Immunosenescence is implicated in aging-related decline and disease and can be shaped by physical and social environments. However, whether early life trauma is directly associated with immune aging in later life remains unclear. Using 2016 venous blood data from the U.S. Health and Retirement Study (HRS), we examined associations between early life trauma and five markers of immunosenescence among adults aged 60 + (n = 8,373). We estimated gender-stratified linear regression models to assess differences in standardized immune measures across trauma exposure categories (0, 1–2, 3, 3+ traumas before age 18). Strikingly, significant associations were observed only among women, and only for those experiencing 3+ traumas compared to none. For instance, women reporting 3+ traumas had a 0.19 higher standardized value of CD4+ EMRA:Naïve (95% CI: 0.08, 0.14) and a 0.16 higher CD8+ EMRA:CD4+ Naïve ratio (95% CI: 0.05, 0.27), adjusting for age. Results were robust to adjustment for trauma in other life stages and parental education, supporting a direct association. Importantly, evidence also suggests a threshold effect: we observed no significant associations when comparing individuals with 1–2 or 3 traumas to those with none, but differences were evident when comparing individuals with 3+ traumas to all other groups, including those with fewer traumas. These findings highlight a threshold beyond which early trauma exerts lasting biological consequences for immune aging. Given the critical role of immune function in healthy aging, our results underscore the importance of early life social environments as key determinants of later-life immune health.

